# MicroRNAs: master regulators in host–parasitic protist interactions

**DOI:** 10.1098/rsob.210395

**Published:** 2022-06-15

**Authors:** Maura Rojas-Pirela, Diego Andrade-Alviárez, Lisvaneth Medina, Christian Castillo, Ana Liempi, Jesús Guerrero-Muñoz, Yessica Ortega, Juan Diego Maya, Verónica Rojas, Wilfredo Quiñones, Paul A. Michels, Ulrike Kemmerling

**Affiliations:** ^1^ Instituto de Ciencias Biomédicas, Facultad de Medicina, Universidad de Chile, Santiago de Chile 8380453, Chile; ^2^ Instituto de Biología, Pontificia Universidad Católica de Valparaíso, Valparaíso 2373223, Chile; ^3^ Facultad de Farmacia y Bioanálisis, Universidad de Los Andes, Mérida, Venezuela; ^4^ Laboratorio de Enzimología de Parásitos, Facultad de Ciencias, Universidad de Los Andes, Mérida, Venezuela; ^5^ Núcleo de Investigación Aplicada en Ciencias Veterinarias y Agronómicas, Facultad de Medicina Veterinaria y Agronomía, Universidad de Las Américas, Chile; ^6^ Centre for Immunity, Infection and Evolution and Centre for Translational and Chemical Biology, School of Biological Sciences, The University of Edinburgh, Edinburgh EH9 3FL, UK

**Keywords:** microrna, host–parasite interactions, kinetoplastids, apicomplexan, diagnostic and therapeutic tools

## Abstract

MicroRNAs (miRNAs) are a group of small non-coding RNAs present in a wide diversity of organisms. MiRNAs regulate gene expression at a post-transcriptional level through their interaction with the 3′ untranslated regions of target mRNAs, inducing translational inhibition or mRNA destabilization and degradation. Thus, miRNAs regulate key biological processes, such as cell death, signal transduction, development, cellular proliferation and differentiation. The dysregulation of miRNAs biogenesis and function is related to the pathogenesis of diseases, including parasite infection. Moreover, during host–parasite interactions, parasites and host miRNAs determine the probability of infection and progression of the disease. The present review is focused on the possible role of miRNAs in the pathogenesis of diseases of clinical interest caused by parasitic protists. In addition, the potential role of miRNAs as targets for the design of drugs and diagnostic and prognostic markers of parasitic diseases is also discussed.

## Graphical abstract

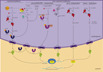

## 1. Introduction

Ribonucleic acids (RNAs) constitute one of the four most abundant macromolecules in mammalian cells. RNA molecules vary in their cellular functions and length (from less than 20 to thousands of nucleotides) [1]. In the past decades, studies about RNA have been focused mainly on coding RNA—messenger RNA (mRNA)—as compared to non-coding RNAs (ncRNAs): ribosomal RNA (rRNA) and transfer RNA (tRNA). However, genome analysis shows that three-quarters of the human genome is capable of being transcribed into RNA. Notably, only 1.5% of these RNAs are protein encoders, the rest being considered ‘non-coding’ [2]. The ncRNAs are a large group of RNA molecules that do not encode proteins but have specialized cellular and molecular functions and are classified into housekeeping and regulatory ncRNAs. The housekeeping ncRNAs are composed of small nuclear RNAs (snRNAs), tRNAs and rRNAs, and their function seems clear. However, the exact function of regulatory ncRNAs, including long non-coding RNAs (lncRNAs), microRNAs (miRNAs) and circular RNAs (circRNAs), is far from being fully understood [3].

Nevertheless, regulatory ncRNAs play essential roles in regulating gene transcription and translation, protein scaffolding, post-transcriptional modification and the establishment of the epigenetic landscape, among others. Thus, they regulate cellular processes such as apoptosis, motility and cell differentiation, which in turn are related to immune responses, and therefore to the pathogenesis and progression of infectious diseases [2,4–9].

The ncRNAs are classified into small ncRNAs with size less than 200nt (e.g. miRNA, piRNA; siRNA) and long ncRNAs with size greater than or equal to 200nt (e.g. lincRNA, NAT) [3,4]. Currently, approximately 8800 small RNAs are annotated by GENCODE; 85% of them are snRNAs, small nucleolar RNAs (snoRNAs), tRNAs and miRNAs [10].

MiRNAs are the most studied ncRNAs, but most of the knowledge of miRNA biogenesis and functions was generated from mammalian systems. The latest release of miRBase (v22) contains sequences from 271 organisms, including approximately 1900 human miRNA precursors, of which at least 725 are highly trusted identifications [11,12]. Furthermore, miRNAs are considered excellent clinical biomarkers since they are much more stable in circulation than other classes of nucleic acids [13]. Particularly in infectious diseases, miRNAs play essential roles in the pathogenesis and innate and adaptive immune responses [14,15].

Here, we will address biological aspects of microRNAs, emphasizing their possible role as regulators of gene expression during pathogen–host interactions. The review is focused on both host and parasites but emphasizes the immune response against kinetoplastid and apicomplexan parasites.

## 2. MicroRNAs

MiRNAs have an average length of 18–25 nucleotides and act as guide molecules in post-transcriptional regulation of genes by base-pairing with the target mRNAs, usually in the 3′ untranslated regions (UTR) of target mRNAs to induce mRNA degradation and translational repression. However, there are reports of interactions of miRNAs with other regions, including the 5′ UTR, coding sequence and gene promoters. Additionally, under certain conditions, these miRNAs can activate translation or regulate the transcription of various types of genes [12,16,17].

The miRNAs gene are present throughout the genome; based on their position, they are categorized into intronic and intergenic subtypes [18]. Intragenic miRNAs genes are found within host genes in either intronic or exonic regions, have independent transcription units, do not overlap with other genes, and their promoter and terminator units regulate their expression [19].

Intronic miRNAs are found in the introns of protein-coding genes and other non-coding RNAs genes [20–22]. They have regulatory elements including promoter-like elements, CpG islands, expression sequence tags and transcription factor-binding sites [21] and are transcribed by RNA polymerase II (pol II) and III (pol III) [23,24]. Most intragenic miRNAs are encoded by polycistronic transcription units that generate multiple miRNAs [18]. Their expression is usually coordinated with their host gene, implying that these miRNAs and host gene mRNAs may be derived from a common precursor transcript [25].

## 3. Location and organization of the miRNA genes

MiRNAs are usually clustered in discrete loci in the genome; both intronic and intergenic miRNAs are present as single or groups of RNAs [26,27]. The latter composed of two or more miRNA genes transcribed from adjacent sequences in the same orientation. In humans, the miRNA clusters are distributed over different chromosomes (Chr), where ChrX, Chr1, Chr13, Chr14, Chr17and Chr19 are the ones that host the highest number of miRNA clusters [27,28]. Furthermore, the number of intergenic miRNA clusters are more numerous than intragenic ones (76 versus 65 miRNA clusters), suggesting that the intergenic region is essential for controlling gene expression [27]. Also, in other organisms such as nematodes, insects, birds and protists, miRNAs are concentrated in specific chromosomes [26].

MiRNA clusters are classified into homo- and hetero-clusters. **The homo-clusters** are composed of miRNAs of the same family, and the control of their targets shows a direct one-stage rapid regulatory coordination [27]. The miRNAs of homo-cluster commonly have an identical ‘seed sequence’ and share a high degree of sequence identity leading to functional redundancy [29,30].

On the other hand, **hetero-cluster miRNAs** are composed of different families of miRNAs involved in complex biological processes, implying more reactions than those regulated by homo-clusters [27,31]. Thus, the miRNAs exert their regulation indirectly through at least three steps, and many of the regulated genes are involved in long-term effects [31].

Many clustered miRNAs are often transcribed in a polycistronic manner [25,32], like the operon regulatory systems present in prokaryotes [33]. The co-expressed miRNAs genes occur at a distance of 50 kb, implying that miRNAs whose genes are separated by less than 50 kb typically derive from a common transcript [25]. Additionally, the expression of these clusters is species- and tissue-specific [34]. For instance, in humans, the 19-miRNA cluster (C19MC), located on chromosome 19, is exclusively found in primates and under physiological conditions only expressed in the placenta and undifferentiated embryonic stem and germ cells [35,36].

The exact origin of the miRNA clusters is unclear; however, *de novo* hairpin birth, duplication processes, insertions, cluster fission, deletions and new miRNAs acquisition, followed by neofunctionalization, are responsible for the evolutionary emergence of miRNAs clusters [27,37–40].

## 4. Biogenesis of miRNAs

**Figure 1.**
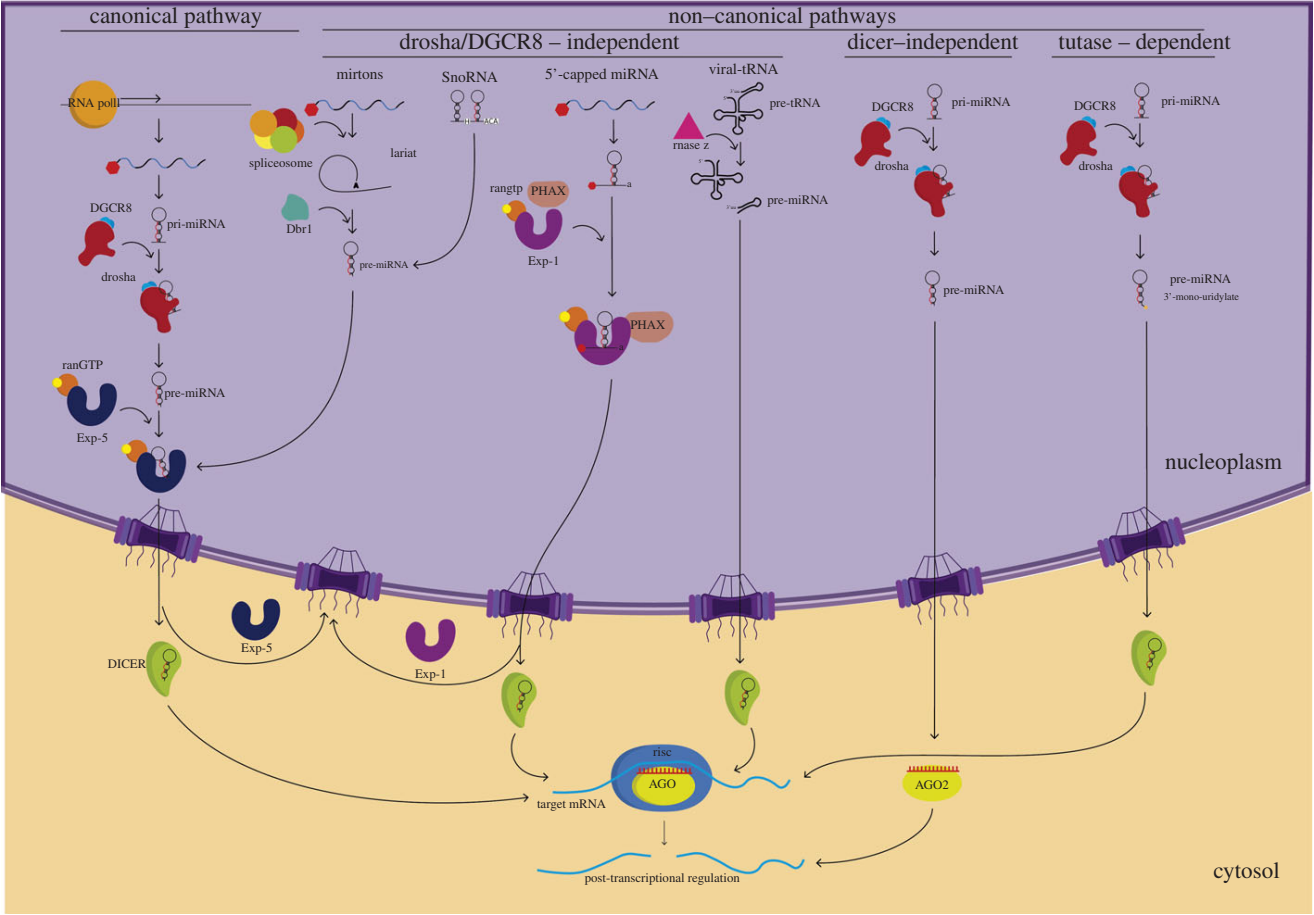
MicroRNA biogenesis pathways. (*a*) Canonical pathway: miRNAs are synthesized by RNApolII and processed in the nucleus by the microprocessor complex (Drosha-DGCR8). Pre-miRNA is exported to the cytosol through Exportin-5 (Exp-5)-RanGTPase. In the cytoplasm, DICER processes miRNAs to bind to the RISC-AGO. (*b*) Non-canonical pathways: miRNAs are processed through microprocessor-independent, TUT-dependent or DICER-independent pathways. In the cytoplasm, all of them are modified by DICER to bind to the RISC-AGO complex, except for DICER-independent processing that binds directly to AGO2 in the cytosol.

The biogenesis of miRNAs begins after their transcription by Pol II or III, generating primary miRNA (pri-miRNAs) with a hairpin structure. These can be processed through different pathways: (i) the canonical pathway and (ii) non-canonical pathways [17,41,42] (figure 1), which are reviewed extensively elsewhere [43–47].

### 4.1. Canonical pathway

The canonical pathway is the most widely used route for processing miRNAs. In this pathway, the pri-miRNAs transcribed by the Pol II are processed, in the nucleus, by a heterotrimeric complex, the Microprocessor Complex (approx. 364 kDa), formed by a ribonuclease III enzyme, Drosha, and two RNA-binding protein DiGeorge Syndrome Critical Region 8 (DGCR8) molecules [48], resulting in a 70–100 nucleotides stem-loop structures called **pre-miRNAs [**49] (figure 1). **DGCR8** recognizes the pre-miRNAs, and **Drosha** serves as a ruler by recognizing basal elements to cleave a distance of 11 base pairs (bp) from the basal junction of single-stranded RNA of double-stranded RNA (ssRNA-dsRNA).

In humans and *Caenorhabditis elegans*, in addition to Drosha and DGCR8, other proteins and auxiliary factors are necessary for pri-miRNA processing. Thus, DEAD-box RNA helicases p72 (DDX17) and p68 (DDX5) interact with the microprocessor complex and facilitate the processing of a subset of pri-miRNAs [50–52]. Moreover, splicing factors (such as SRp20) enhance the processing of pri-miRNA [53].

The pre-miRNAs associate with the **exportin 5 (Exp-5) / guanine triphosphatase (GTPase) Ran (RanGTP) complex** (figure 1). Exp-5 translocates the pre-miRNA from the nucleus to the cytoplasm and protects the pre-miRNAs from nuclease degradation [54,55]. RanGTP provides the necessary energy and helps stabilize the interaction of Exp-5 with the pre-miRNA [54]. In the cytoplasm, the pre-miRNA is released from the **Exp-5-RanGTP-pre-miRNA heteroternary complex** through the Ran-binding proteins (RanBPs) by inducing a conformational change of RanGTP [56].

In the cytoplasm, the pre-miRNAs undergo cleavage by the RNAse III-like endonuclease Dicer, along with dsRBD, transactivation response element RNA-binding protein (TRBP), protein kinase RNA activator (PACT) and loquacious-PD [57,58] (figure 1). The processing by the **Dicer-dsRBD-binding protein complex** involves the removal of the terminal loop, resulting in a mature approximately 22 nt-long miRNA duplex (miRNA/miRNA*), which is made up of a guide chain (miRNA) and a passenger chain (miRNA*).

The miRNA/miRNA* is then loaded into the Argonaute (AGO) family of proteins through ATP-dependent chaperone proteins (HSC70/HSP90) [12,59,60] promoting the expulsion and degradation of the miRNA* [59,61] and the formation of the **RNA-induced silencing complex (RISC)**. RISC recognizes the targeted mRNA through base-pairing with miRNA [60] (figure 1).

### 4.2. Non-canonical pathways

Multiple pathways of alternative non-canonical miRNA biogenesis are responsible for synthesizing diverse small RNAs structurally and functionally like miRNAs. Although they are characterized by presenting various combinations of the different proteins involved in the canonical pathway (such as Drosha, Dicer, AGO and Exp-5), they bypass one or more steps observed in the canonical biogenesis pathway or include additional steps in the miRNA maturation process. These non-classical biogenesis pathways are classified into three groups: Drosha / DGCR8-independent, Dicer-independent and terminal uridylyl transferases (TUT) dependent [12,17,62–64] (figure 1).

#### 4.2.1. Drosha/DiGeorge Syndrome Critical Region 8 (microprocessor)-independent pathways

The Drosha-independent pathways generate pre-miRNA-like hairpins that serve as Dicer substrates.

##### 4.2.1.1. Mirtrons

In this pathway, mirtrons (18% of the total miRNA population) are generated by the spliceosome and the lariat-debranching enzyme DBR1 [65] (figure 1). The mirtron hairpins access the canonical miRNA pathway during nuclear export, where they are processed by Dicer and incorporated into RISC [66].

##### 4.2.1.2. 5′-capped microRNAs or exportin-1-dependent microRNAs

The pre-miRNAs are generated directly through the transcription of RNA Pol II and (figure 1) are 7-methylguanosine (m7G)-capped at their 5′ ends, while the 3′ ends are produced by transcription termination. Unlike canonical miRNAs, this m7G-capped pre-miRNA uses the PHAX (phosphorylated adapter for RNA export)-dependent Exportin-1 (XPO1) pathway for nuclear-cytoplasmic transport [67]. Once in the cytoplasm, the m7G-capped pre-miRNA is processed by Dicer [47].

##### 4.2.1.3. Small nucleolar RNAs-derived microRNAs pathway

snoRNAs [44,68,69] are highly conserved nucleolar non-protein-coding RNAs synthesized by an intron-process [70–74].

In protists such as *Giardia lamblia* (*G. lamblia*), six sno-miRNAs have been identified and constitute 4.8% of the miRNA pool [75–77]. Furthermore, sno-miRNA-2, a Dicer-digested product from GlsR17 snoRNA, has putative target sites at the 3′-UTRs of many variant surface protein (VSP) mRNAs. Thus, the regulation of the differential expression of distinct VSPs through sno-miRNAs during the cycle of infection may be a mechanism that contributes to the pathogenicity of *Giardia* [75,77].

##### 4.2.1.4. tRNA viral- derived microRNAs

Different γ-herpes viruses encode multiple miRNAs [78,79] that are co-transcribed downstream of tRNAs [79–81] by RNA pol III and [46,80,81] cleaved by a cellular ribonuclease Z (RNaseZ) at the 3′ end of the tRNA to liberate pre-miRNA hairpins, which in turn are processed by Dicer to yield the mature viral miRNAs [46,81] (figure 1).

#### 4.2.2. Dicer-independent pathways

The biogenesis of certain miRNAs does not require Dicer and instead involves the catalytic activity of AGO2 protein [82–84] (figure 1). Thus, the pre-miRNAs are processed by Drosha, but are cleaved by the AGO2 catalytic centre, followed by resectioning its 3′ terminus [83–86]. Structural analyses indicate that those miRNAs have features that differentiate them from other ‘canonical’ miRNAs. Thus, the conserved 42-nt hairpin is too short to be efficiently recognized and processed by Dicer [83,87].

#### 4.2.3. Terminal uridylyl transferases-dependent pathways

This pathway is very similar to the canonical miRNA biogenesis pathway. However, it has an additional step, including interaction with TUT [64,88] (figure 1) that function as an integral regulator of the biogenesis and specifically mono-uridylate the 3′ ends of the pre-miRNAs, yielding the two-nucleotide 3′ overhangs required for efficient Dicer-mediated processing [64,88].

## 5. Subcellular localization of miRNAs and attribute functions

**Figure 2.**
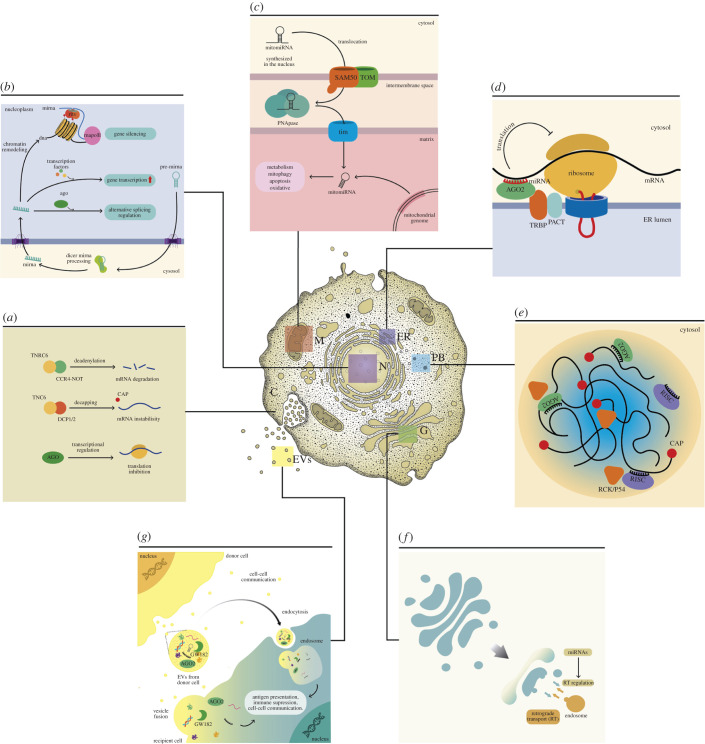
MicroRNAs’ location and functions in the cell. (*a*) Cytoplasm: post-transcriptional silencing in the cytoplasm is the classic function mediated by miRNA via RISC. (*b*) Nucleus: regulatory mechanisms of nuclear miRNAs. (*c*) Mitochondria: mitochondrial miRNA (MitomiR)-targeting mitochondrial mRNAs. MiRNAs are synthesized in the nucleus (mitomiRNAs) and are imported into the mitochondria. (*d*) Endoplasmic reticulum: repression of translation occurs at MBP. (*e*) P-bodies: miRNAs are involved in P-bodies formation. (*f*) Golgi apparatus: MiRNAs are involved in resistance to and trans-Golgi or the RT. (*g*) Extracellular vesicles: MiRNA in cell-to-cell communication. EVs released by donor cells can fuse directly with the plasma membrane of recipient cells to discharge their content.

MiRNAs are located in the cytoplasm, the nucleus and other cell compartments, and the extracellular medium [89–91] (figure 2).

### 5.1. Intracellular miRNAs

#### 5.1.1. Cytoplasm

MiRNAs act mainly in the cytoplasm, where they represent 53% of small RNAs [92], but most of them have dual subcellular locations [93]. In the cytoplasm, miRNAs are focused on post-transcriptional silencing through three main pathways, all of which involve the recruitment of protein complexes that alters mRNA stability (figure 2*a*): (i) through the recruitment of the trinucleotide repeat-containing gene 6A protein (TNRC6), which together with CCR4-NOT deadenylase complex, leads to deadenylation and degradation of mRNA; (ii) through TNRC6 mediated recruitment of Dcp 1/2 decapping enzyme complex, which cleaves 5′ cap of mRNA promoting mRNA destabilization; and (iii) through direct binding of AGO2 to mRNA inhibiting translation [94].

#### 5.1.2. Nucleus

Although the canonical biogenesis of miRNAs begins in the nucleus and ends in the cytoplasm, miRNAs can return to the nucleus. Thus, many miRNAs have a dual subcellular location [95], where approximately 75% of cellular miRNAs are present in the nucleus and the cytoplasm [96]. The exact role of nuclear miRNAs is unclear; however, it has been suggested that they act in post-transcriptional gene silencing (TGS) through a nuclear RISC-dependent mechanism (nRISC) [95] (figure 2*b*). Alternatively, miRNAs may play a key role in epigenetic regulation inducing TGS by recruiting the RNA-induced transcriptional silencing complex to specific DNA sites (figure 2*b*) causing alterations in the chromatin structure through CpG methylation [95,97]. On the other hand, miRNAs are also associated with transcriptional gene activation by recruiting transcriptional activators [98] by promoting lncRNA silencing, acting as a ‘repressor of a repressor’ [99].

Notably, the miRNA-AGO complex may play a key role in regulating alternative splicing [95,100] (figure 2*a*). Thus, chromatin remodelling in corresponding regions with introns disrupts the splicing of adjacent exons. Furthermore, the regulation of the state of packing of chromatin-mediated by miRNA in the target exon can affect the speed of Pol II processing [100].

#### 5.1.3. Mitochondria

It is estimated that between 6 and 27% of the human miRNA can be detected in the mitochondria [101,102]. They are imported from the cytoplasm and processed in the mitochondria for post-transcriptomic regulation of mitochondrial and nuclear genes [103,104]. The mitomiRs cross the mitochondrial membrane through the transport systems SAM50, mitochondrial translocases of the outer membrane and the inner membrane (figure 2*c*). Furthermore, the PNPase enzyme, a 3′, greater than 5′ exoribonuclease and poly-A polymerase, in the mitochondrial intermembrane space, may also help to import the miRNAs into the mitochondria [105]. In addition, mitomiRs could also be synthesized in the mitochondrial matrix and processed by endogenous DICER.

Although the regulation of gene expression is usually observed as a downregulation [106], it has been observed that mitomiRs increase the translation of some miRNAs [107]. The downregulation of mitochondrial genes occurs mainly through mRNA degradation, decapping and deadenylation [104]. The translational enhancement requires specific miRNA for mRNA base-pairing and AGO2 binding [107]. Importantly, some mitomiRs target nuclear-encoded mRNAs localized on the mitochondrial surface [102]. Notably, it has been suggested that some mitomiRs are encoded in the mitochondrial DNA (mtDNA) [103] (figure 2*c*) and might originate from mtDNA-derived mRNA molecules.

MitomiRs are associated with different mitochondrial processes, including metabolism, apoptosis, mitophagy, oxidative stress pathways and structure [101,108] (figure 2*c*).

#### 5.1.4. Endoplasmic reticulum

MiRNAs are present in the rough RER and act as a site for the repression of mRNA translation [109–111] (2D). The miRNA-mediated repression occurs in membrane-bound polysomes (MBP) and requires the Altered Meristem Program1 (AMP1) protein.

In mammalian cells, TRBP and PACT have been identified as key factors anchoring RISC to Endoplasmic reticulum (ER) membranes in an RNA-independent manner [112]. Thus, the newly formed target mRNA is located first in the ER-linked polysomes, then the binding of the miRNA / AGO2 complex occurs, and finally, the translation is repressed [110] (figure 2*d*).

Interestingly, the mitochondria regulate AGO2–miRNA complex formation in polysomes attached to the REa. Thus, alterations in mitochondria's functioning (as depolarization) and morphology impair the endosome—ER interaction, causing a downregulated miRNA / AGO2 complex turnover [113]. Kinetoplastids, such as *Leishmania donovani,* induce depolarization of the mitochondria in host cells to manipulate the miRNA network [114]. However, this will be further discussed in later sections.

#### 5.1.5. Processing bodies

Processing bodies (P-bodies) are cytoplasmic ribonucleoprotein (RNP) granules with roles in post-transcriptional regulation, highly conserved in eukaryotes. These structures are dynamically formed during the cell cycle in response to extracellular signals and are composed of translationally repressed mRNAs and proteins [115]. In addition, AGO proteins interact in P-bodies with RNA helicases and other proteins to inhibit protein synthesis or promote mRNA degradation [90,116,117].

In addition, miRNAs are necessary for the integrity of the P bodies. It has been postulated that the P-bodies arise as a consequence of the siRNA, dsRNA and miRNAs-mediated silencing [118], and they are probably the primary storage site of translationally repressed mRNA [90] (figure 2*e*).

On the other hand, the RISC complex affects translation through direct inhibition of translation initiation and the formation of the P- bodies. Thus, the interaction between the AGO–miRNA complex and its target, the assembly of an mRNA/Protein (mRNP) complex, leads to the direct location of the mRNA inside the P-bodies, preventing protein synthesis [119].

#### 5.1.6. Golgi apparatus

The function of miRNAs in the Golgi apparatus has been less studied. Nonetheless, some reports have documented their relationship with the functions of this organelle and the resistance to chemotherapeutic drugs in cancer cells, psychiatric disorders and regulation of retrograde transport (RT) [120–122] (figure 2*f*).

The RT is a highly selective pathway that allows receptors and other molecules internalized by cells to be delivered to the trans-Golgi network and is regulated by miRNAs (figure 2*f*) [121,123].

### 5.2. Extracellular miRNAs

MiRNAs have also been identified in the extracellular space. Particularly miRNAs and RISC components (AGO2 and GW182) have been identified in extracellular vesicles (EVs) [124] where they can carry out their conventional function or act as a ligand for toll-like receptors (TLRs) present on the surface of immune cells [125,126] (figure 2*g*). Thus, antigen presentation, activation, surveillance, immune suppression and intercellular communication are regulatory mechanisms affected by EVs [127]. On the other hand, the fact that circulating miRNAs can be transferred from one cell to another and bind to receptors could attribute a hormone-like function to them. In this case, these miRNAs could be called hormone miRNAs or H-miRNAs [125,128].

In addition, in parasitic diseases caused by helminths and apicomplexans, EV-derived miRNAs play a crucial role in host–parasite interaction [129,130] and act as modulators of drug sensitivity [131]. However, this will be discussed further in-depth in the following sections.

## 6. MicroRNAs in the host–parasite interaction

Parasites and their hosts have co-evolved in an intricate relationship to establish chronic infections using evasion mechanisms that serve to avoid and regulate the host's defence mechanisms. In this context, miRNAs are ideal tools for parasites to modulate gene expression in host cells since miRNAs are non-immunogenic, can be transported and transferred in exovesicles from the pathogen to the host cells and can evolve rapidly to target new transcripts [132]. Thus, miRNAs are crucial during host–parasite interactions. For instance, in the host cells, miRNAs can favour the elimination of the pathogen [133,134], while in the parasites, they regulate different physiological processes such as developmental transition, sexual reproduction, expression of antigenic molecules, and virulence factors, promoting the parasites' subversion strategy and survival [134–137]. Thus, different pathogens induce a miRNA-mediated post-transcriptional regulation of genes involved in the inflammatory and immune responses [138], cell cycle, apoptosis, autophagy and cytoskeleton reorganization [138,139]. Moreover, intracellular pathogens modulate their own and host miRNAs that participate in cellular processes relevant to pathogen replication and promotion of its life cycle [140]. Particularly interesting are miRNAs delivered by exovesicles since they are mediators of Inter-Kingdom communication between host cells and pathogens [141,142].

Therefore, miRNAs might actively change the outcome of infections. Host miRNA dysregulation has been associated with impaired immune response and increased host colonization by the pathogen. Contrarily, host miRNAs can be part of the host's mechanism of defence against the parasites. Besides their potential as diagnostic and prognostic tools, miRNAs are potential targets for chemo and immunotherapies for parasitic diseases [142].

In the following section, we review the role of miRNAs in infections caused by kinetoplastids and apicomplexan parasites.

### 6.1. miRNAs in infections caused by kinetoplastids

#### 6.1.1. miRNAs in *Trypanosoma cruzi*–host interactions

**Figure 3.**
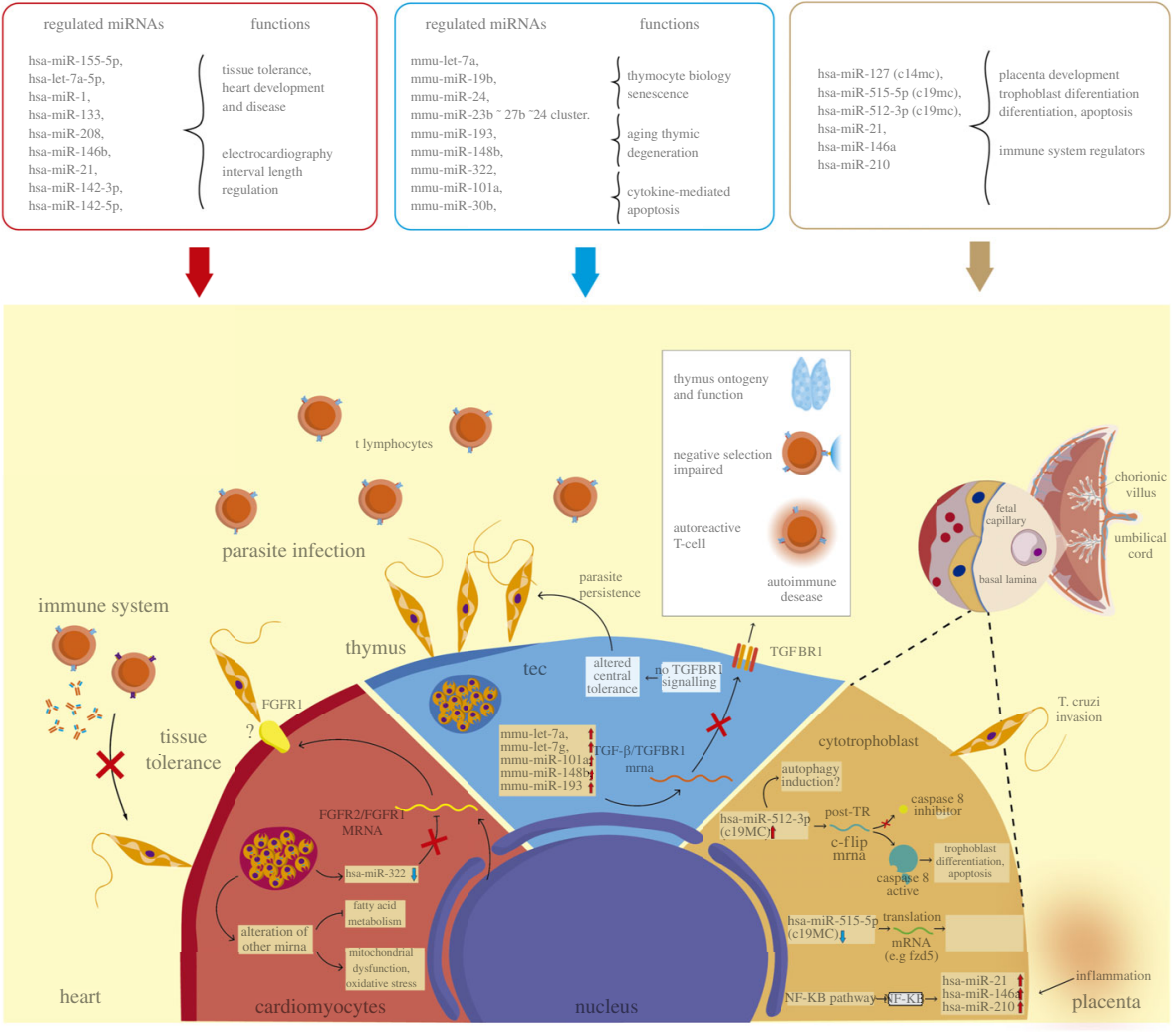
MicroRNAs in *T. cruzi*–host interaction. Regulatory mechanisms of miRNA genes in different organs during *T. cruzi* pathogenesis. Cell diagram: cardiac cells (Red portion). *T. cruzi* infection induces in cardiomyocytes the dysregulation of some miRNAs involved in regulating fatty acid metabolism and some pathways related to the immune response during pathogenesis. The downregulation of some miRNAs, such as hsa-miR-322, positively regulates the expression of FGF2 and FGFR1 mRNA. The products of these genes, FGF2 and FGR1 proteins, are incorporated into the host cell's plasma membrane and could be a possible target for parasite–host interaction. Additionally, infection by *T. cruzi* can induce tolerance. Thymus cell (Blue portion). Parasite infection causes upregulation of some miRNAs, including mmu-let-7a, mmu-let-7 g, mmu-miR-101a, mmu-miR-148b and mmu-miR-193 in.TEC. Several of these miRNAs repress the expression of TGF-β and its receptor TGFBR1, impairing the normal function of the thymus and thus affecting some processes such as ontogeny, negative selection and the accumulation of autoreactive lymphocytes involved in autoimmune diseases. Placenta cells (brown portion). *T. cruzi* infection of cytotrophoblasts induces the dysregulation of C19MC miRNAs and immunomiRs. The upregulation of hsa-miR-512-3p is responsible for activating the caspase 8 pathway shown to be involved in trophoblast differentiation and apoptosis mechanisms. The upregulation of this miRNA is possibly associated with the autophagy induction in the infected cell. Also, hsa-miR-515-5p downregulation allows the expression of some genes crucial for the differentiation of human trophoblasts during *T. cruzi* infection. Under an inflammatory environment, the upregulation of immunomiRs (hsa-miR-21, hsa-miR-146a and hsa-miR-210) occur, influenced by the NF-kB pathway.

During *Trypanosoma cruzi* infection, changes in gene expression occur in both the parasite and the host cell. In the host cell, both protein-coding and non-coding genes alter their expression in response to the presence of the parasite [138,143,144]. Although *T. cruzi* lacks canonical miRNA-induced silencing mechanisms [145], infection by this parasite induces changes in the expression of miRNAs in the host cell (figure 3). The type of miRNA altered depends on the cell type and even on post-infection time [146,147].

Several miRNAs are involved in the pathogenesis of chagasic cardiopathy during acute and chronic *T. cruzi* infection [146,148,149] (figure 3). In response to infection, the expression of up to 133 miRNAs is altered [149], including hsa-miR-155-5p, hsa-let-7a-5p, the muscle, and myocardial-specific hsa-miR-1, hsa-miR-133 and hsa-miR-208 [148,149], that are involved in tissue tolerance and heart development [150–152] (figure 3). In addition, hsa-miR-146b, hsa-miR-21, hsa-miR-142-3p and hsa-miR-142-5p expression correlates with clinically relevant parameters such as parasitemia and electrocardiography changes (QTc interval) [149] (figure 3). Thus, miRNAs that are related to electrocardiographic changes regulate the CACNA1C (Calcium channel), GJA5 (Gap Junction Protein, alpha 5), RNF207 (Ring finger protein 207) and KCNA1 (potassium voltage-gated channel shaker-related subfamily, member 1) and SLC18A2 (Solute carrier family 18 members 2) genes [149]. In addition, has-miR-21 is essential for controlling and balancing initial pro-inflammatory and later immunoregulatory and anti-inflammatory responses in the heart [153]. At the same time, has-miR-146a/b modulates the TLR signalling cascade and immune tolerance [154,155].

Interestingly, hsa-miR-322, hsa-miR-139-5p, hsa-miR-145 and hsa-miR-149 are always downregulated throughout the infection process [149]. Although these miRNAs are not specific to cardiac tissue, they have been attributed essential roles in cardiac protection [156–159]. For instance, hsa-miR-322 regulates the insulin pathway and has a cardioprotective effect in a model of metabolic syndrome [156] and against ischaemia/reperfusion-induced injury [160]. Thus, has-miR-322 regulates the expression of fibroblast growth factor 2 (FGF2) and its fibroblast growth factor receptor 1 (FGFR1) [161], the principal FGF receptor in the heart, which both are involved in cardiac fibrosis and cellular conduction [162]. In addition, the FGFR1 receptor is used by some pathogens, such as *Neisseria meningitidis* and rickettsia, for internalization into host endothelial cells (ECs) [163,164]. Thus, the downregulation of hsa-miR-322 in.response to *T. cruzi* infection could increase FGF2 / FGFR1 and play an important role in the invasion process (figure 3).

Other miRNAs are differentially expressed during chagasic cardiac disease progression. Thus, hsa-miR-238-3p, hsa-miR-149-5p, hsa-miR-143-3p, hsa-miR-145-5p, hsa-miR-486-5p, hsa-miR-138-5p, hsa-miR-9-5p, hsa-miR-26a-5p, hsa-miR-185-5p, hsa-miR-200b-3p and hsa-miR-335-5p target genes related to arrhythmia, fibrosis, myocarditis and hypertrophy [146].

*Trypanosoma cruzi* infection induces progressive thymic atrophy or involution [165–169]. In addition, the parasite causes loss of immature CD4^+^ CD8^+^ thymocytes due to premature apoptosis [167], phenotypic and functional disturbance of the regulatory T cell population [169], thymocyte migratory disturbances [165,168] and neuroendocrine-immune imbalance [165–167]. Furthermore, it has been proposed that miRNAs are involved in thymic involution [170] since *T. cruzi* modulates the expression of at least 29 miRNAs in medullary and cortical thymic epithelial cell (TEC) populations. Thus, mmu-let-7a, mmu-miR-19b, mmu-miR-193, mmu-miR-101a, mmu-miR-30b, mmu-miR-148b, mmu-miR-322, mmu-miR-24 and mmu-miR-23b–27b–24 clusters (figure 3) were upregulated; however, the expression pattern of these miRNAs is dependent on whether TEC exhibited a cortical or medullary phenotype [170].

On the other hand, hsa-miR-24 is related to signalling pathways of senescence [171], hsa-miR-193, hsa-miR-148b and hsa-miR-322 are related to age-related thymic involution [172,173], and hsa-miR-101a and hsa-miR-30b modulate cytokine-mediated cellular apoptosis and dysfunction [174]. Notably, many of the 29 differentially expressed miRNAs target genes related to chemotaxis, cell adhesion and inhibition of apoptotic externals signals [170]. Interestingly, the *T. cruzi*-induced mmu-let-7a, mmu-let-7 g, mmu-miR-101a, mmu-miR-148b and mmu-miR-193 have in common that they target the transforming growth factor-β gene (TGF-β) and the gene for its receptor [175–178] (figure 3). In the thymus, TGF-β signalling is crucial not only for thymus ontogeny and function [179] but also for the negative selection of T cells, and therefore self-tolerance [180]. Thus, the alteration of self-tolerance by the parasite could be a mechanism to promote persistent infection [181] (figure 3). Although the thymus may be already atrophied during *T. cruzi* infection, this mechanism still could allow the antigen presentation to recycle memory parasite-specific T cells moving from the periphery to the thymic microenvironment. Thus, the activation of these cells in the intrathymic environment could make them susceptible to clonal deletion [181]. Additionally, the presence of *T. cruzi* antigens in the thymus could lead to the generation of parasite-specific regulatory T cells contributing to a ‘parasite tolerance’ [181].

Notably, some altered miRNAs in response to *T. cruzi* infection are common in cardiac and thymic tissue infections. Hsa-miR-322/mmu-miR-322 (downregulated in cardiac tissue and upregulated in thymic tissue) [149,170] seems to have a dichotomous role in *T. cruzi* infection. It seems necessary to establish the infection, although it appears to be modulated depending on the tissue/cell. In the thymus, the upregulation of mmu-miR-322 would be more associated with the modulation of cellular metabolism. The overexpression of this miRNA induces an increase in the expression of genes related to the metabolism of fatty acids (FAs) [156]. While *T. cruzi* has the ability for FA synthesis, it has been found that intracellular amastigotes may depend on host FA metabolism to support infection [182,183] and take advantage of host cell metabolism [184]. In addition, amastigotes promote FA uptake and oxidization, suggesting a synchronization of parasite growth to FA metabolism in the host [185] [182]. Considering that FAs are an important energy source for thymus cells [186] and that *T. cruzi* is partially dependent on the FA metabolism host, the upregulation of hsa-miR-322 may provide an increase in host fat FA metabolism to their benefit.

Alternatively, due to the stress conditions generated by the presence of the parasite, miRNAs can act as restorers of homeostasis or as enhancers of gene expression programs that allow cell populations to adapt to changes in the thymic microenvironment [170].

In the placenta, miRNAs also have been proposed as key players in defence against *T. cruzi* [187]. The parasite induces, in *ex vivo* infected human placental explants (HPE), differential expression of 14 miRNAs that target genes involved in development, immunity, placenta pathologies and infection [144] (figure 3). Interestingly, the largest miRNA cluster in humans is encoded on chromosome 19 (C19MC) (19q13.41) and is almost exclusively expressed in the placenta [35,36]. Thus, hsa-miR-515-5p and hsa-miR-512-3p are encoded in the C19MC cluster and, respectively, decrease and increase their expression in the presence of the parasite [144] (figure 3). Hsa-miR-515-5p targets the genes that code for aromatase P450 (CYP19A1), frizzled 5 (FZD5) and glial cells missing transcription factor 1 (GCM1), genes that are essential for human trophoblast differentiation [188]. Interestingly, trophoblast differentiation has been described as a possible placental defence mechanism against *T. cruzi* [189–191]. Therefore, it is very likely that the downregulation of hsa-miR-515-5p partially contributes to the parasite-induced trophoblast differentiation. On the other hand, hsa-miR-512-3p regulates the expression of the caspase 8 inhibitor c-FLIP (FLICE-like cellular inhibitory protein) and subsequently promotes caspase 8 activity [192]. Caspase 8 also regulates trophoblast differentiation and apoptotic cell death and is activated by *T. cruzi*, inhibition of this enzyme increases parasite infection in a trophoblastic cell [193] (figure 3). Thus, parasite-induced hsa-miR-512-3p upregulation could also be part of the protective placental response, probably associated with trophoblast differentiation, against *T. cruzi* infection.

Another placenta-specific miRNA is hsa-miR-127; it is encoded in the C14MC cluster [194] and related to placental development [195]. However, its decrease is associated with recurrent miscarriage and the fetus being small for gestational age (SGA). Furthermore, during *ex vivo* infection of HPE with *T. cruzi,* hsa-miR-127 also decreases, and newborns with congenital Chagas disease can present clinical features similar to SGA [144] (figure 3). Moreover, as in cardiac muscle, hsa-miR-21 and hsa-miR-146a were overrepresented in response to *ex vivo* infection in HPE [144]. Finally, it should be noted that the expression of several miRNAs, including hsa-miR-21 and hsa-miR-146a, is influenced by the NF-κB signalling pathway [196] during an inflammatory process response [197]. Interestingly, *T. cruzi* activates NF-kB signalling pathways in HPE [198]. Therefore, the observed increase of hsa-miR-21 and hsa-miR-146a expression might be related to the NF-kB signal transduction pathways (figure 3).

### 6.2. miRNA in host–leishmania interaction

**Figure 4.**
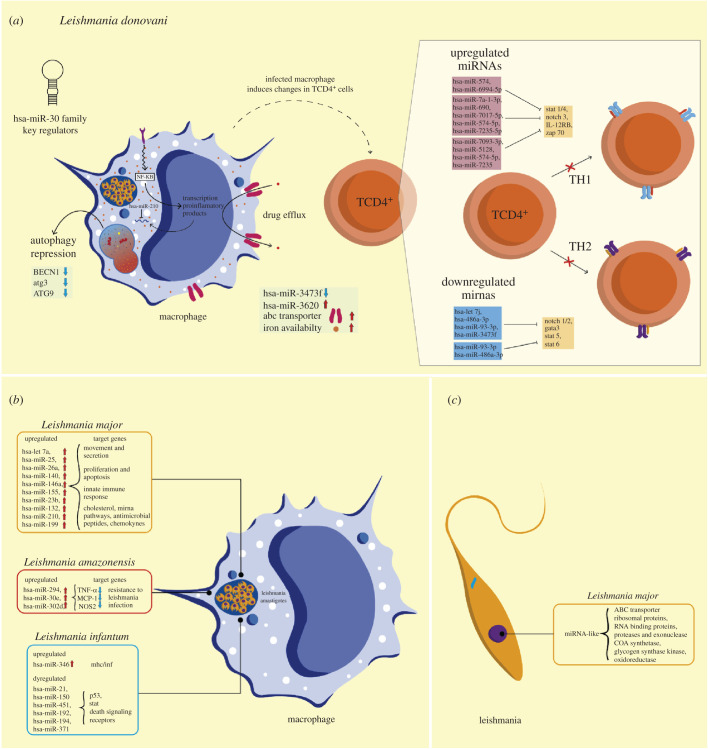
MicroRNAs in leishmania–host interaction. (*a*) miRNAs during *Leishmania donovani* infection. miR-30 family miRNAs govern several processes during *L. donovani* infection of macrophages, including the repression of autophagy-related proteins BECN1, ATG3 and ATG9. Post-transcriptional regulation of inflammatory products by hsa-miR-210 also occurs. Additionally, the accumulation and availability of iron (orange dots) and the overexpression of ABC transporters responsible for drug efflux are also modulated by miRNAs. Alternatively, *L. donovani* infection influences miRNA expression in T cells that compromise cell polarization. (*b*) miRNA dysregulation and target genes during infection of macrophages with different species of leishmania. (*c*) Targets of miRNA-like molecules identified through *in silico* studies in *Leishmania major*.

Leishmania infection alters miRNAs expression in the host cell depending on the parasite and host species. In addition, the leishmania-modulated miRNAs promote the persistence of infection through regulating genes involved in host immune responses [133,199–203] (figure 4).

#### 6.2.1. Leishmania donovani

*Leishmania donovani* infection in CD4^+^ T cells induces a differential expression of 208 miRNAs, most of them of the let-7 family (figure 4*a*). Most of the upregulated miRNAs target transcription factors involved in polarizing TCD4 differentiation to the Th1 phenotype, mainly linked to the IFN-γ pathway [203]. Interestingly, IFN-γ plays a fundamental role in controlling leishmania infection, suggesting that the increase of miRNAs related to the modulation of this cytokine could be a virulence strategy of the parasite [204]. Furthermore, INF-γ is also required for the sustained expression of CXCL10/IP-10 (also known as interferon-inducible protein-10), necessary for developing a robust protective Th1 response [205]. Thus, *L. donovani* may use miRNAs to interfere with the functions of IFN-γ-activated macrophages and ensure their intracellular survival. On the other hand, downregulated miRNAs target transcription factors involved in the differentiation of CD4 T cells in the Th2 population (STAT 5, STAT 6, GATA 3, Notch ½ and Jak1/3) and the production of Th2-type cytokines, such as IL-2, IL-4 and IL-13 [203]. Therefore, these latter miRNAs could favour the preferential differentiation of CD4 T cells into the Th2 phenotype (figure 4*a*), leading to an exacerbated infection.

Other miRNAs, including the miRNA-30 family, hsa-miRNA-3473f, hsa-mir-3620 and hsa-miRNA-3620, are differentially expressed *in vitro* macrophage infection and are involved in the inhibition of autophagy, drug response, hypoxia and iron homeostasis [14,200,202,206] (figure 4*a*). For instance, the miRNA-30 family promotes downregulation of the autophagy-related genes BECN1, ATG3 and ATG9, and subsequently, survival of the parasite. Importantly, cells can also use autophagy to eliminate protist pathogens and the generation of microbial antigens leading to the activation of immunity [206,207]. Upregulation of hsa-miR-3620 seems to regulate iron homeostasis and hypoxia in leishmania-infected cells [200] (figure 4*a*), assuring iron availability in cell cytoplasm for the parasite. In addition, iron is a major factor regulating the transition of promastigotes to amastigotes in leishmania [200,208].

In addition, an EVs-dependent mechanism to regulate host gene expression has been proposed [209]. Thus, *L. donovani* release EVs containing GP63 metalloprotease, which can cleave Dicer 1 in.murine hepatocytes, resulting in downregulation of hsa-miRNA-122 expression and a consequent decrease in serum cholesterol and an increase in murine liver infection [209].

#### 6.2.2. Leishmania major

*Leishmania major* also induces a differential expression in miRNAs in different host cells [133,199,210] (figure 4*b*). Thus, the expression of 365 miRNAs was evaluated in infected macrophages, and about 20% [64] of them were differentially expressed, including hsa-let-7a, hsa-miR-25, hsa-miR-26a, hsa-miR-140, hsa-miR-146a, hsa-miR-155, hsa-miR-23b, hsa-miR-132, hsa-miR-210 and hsa-mir-199 [133,210] (figure 4*b*).

Most of these 64 miRNAs were upregulated during the first 3 h post-infection, where the target genes are involved in cell movement and enzyme production and secretion [133]. Moreover, hsa-miR-146a and hsa-miR-155 are essential for the modulation of both the innate and adaptive immune responses [133,199,210]; interestingly, both miRNAs are also upregulated during *T. cruzi* infection [144,149].

Other targeted genes are involved in proliferation, pro-and antiapoptotic pathways, innate immune response pathways, intracellular cholesterol trafficking, miRNA expression pathways and production of anti-microbial peptides and chemokines [133].

It is important to point out that unlike *L. donovani*, where transcription factors and elements converge, in *L. major,* the INF-γ pathway is the main miRNA target. This might explain the fact that leishmania species are differentially responsive to IFN-γ [204]. *L. major* can survive in an environment enriched with IFN-γ since it can counteract the IFN-γ response in macrophages by modulating genes involved in the innate immune response, cell adhesion and proteasomal degradation [211]. Importantly, like *L. donovani*, *L major* induces the expression of miRNAs that target genes linked to the MAPK signalling pathway [199].

### 6.3. Other leishmania species

*L. amazonesis, L. viannia* and *L. infantum* also modulate miRNA expression to subvert host defences and allow parasite survival and replication [201,212]. Thus, *L. amazonensis* upregulates hsa-miR-294, hsa-miR-30e and hsa-miR-302d, decreasing the expression of TNF-α, monocyte chemoattractant protein-1 (Mcp-1) and oxide synthase 2 (Nos2) [213] (figure 4*b*), which are critical proteins for resisting leishmania infection [213–215]. In macrophages, *L. viannia* and *L. infantum* upregulate hsa-miR-346, modulating MHC- and interferon-associated genes [201]. Studies in dogs, the main reservoir host for *L. infantum* infection, revealed that in peripheral blood mononuclear cells, hsa-miR-21, hsa-miR-150, hsa-miR-451, hsa-miR-192, hsa-miR-194 and hsa-miR-371 were differentially expressed in the presence of this protist [216]. Moreover, only the expression of hsa-miR-150 and hsa-miR-194 is positively correlated with parasitic load [216].

Although in leishmania the RNAi activity has been lost, computational studies have identified miRNA-like elements [210,217] (figure 4*c*). Thus, 25 potential miRNA-like elements coding genes have been identified on different chromosomes of the *L. major* genome [217]. Furthermore, nine of these miRNA-like elements have motif sequence patterns similar to the sequence of human hsa-miR-146a. Therefore, they could be attached to human AGO2 [210]. Notably, the potential target genes of the miRNA-like elements are associated with drug resistance factors (such as ABC transporter, ribosomal protein, RNA-binding proteins, hydrolase and exonuclease) and enzymes related to metabolic pathways (acetyl-CoA synthetase, glycogen synthase kinase and oxidoreductases) [217]. Thus, it is very likely that leishmania can process these miRNA-like elements and use them to mimic miRNAs functions and modulate the immune system [210].

### 6.4. miRNA in apicomplexa infections

#### 6.4.1. miRNA in host–plasmodium interaction

**Figure 5.**
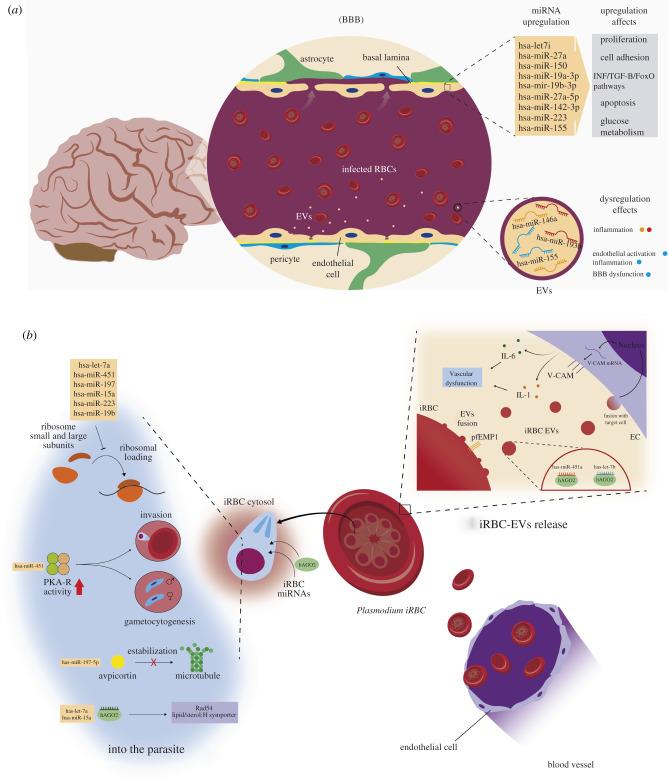

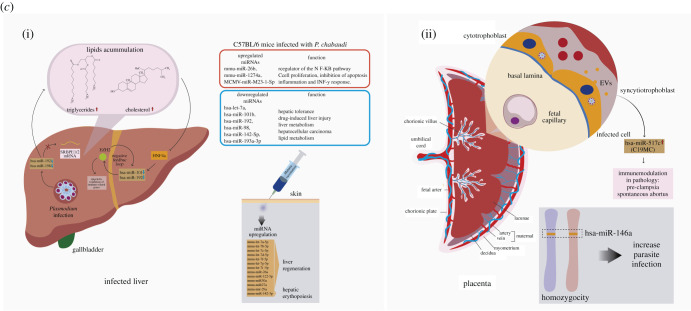
(*a*) MicroRNAs during plasmodium pathogenesis. (*a*) CM. Plasmodium-infected RBCs accumulate in the brain blood vessels and cross the BBB. It causes the alteration of brain cells' microRNAs and their function regarding proliferation and adhesion of cells, signalling pathways, apoptosis and carbohydrate metabolism. On the other hand, the release of EVs by host cells is involved in inflammation and BBB dysfunction, mainly because of hsa-miR-146a, hsa-miR-193b and hsa-miR-155. (*b*). MicroRNAs in NCB. Several events are caused by miRNA dysregulation in the parasite, the erythrocyte and endothelial cells. Inside the parasite, miRNAs affect directly ribosomal loading and translation, invasion process, survival and gametogenesis (hsa-miR-451), but also the microtubules stability (hsa-miR-157-5p) and the parasite replication due to the action of imported human AGO (hAGO2) and the regulation of Rad54 and the Lipid/sterol: H symporter by hsa-let-7a and hsa-miR-15a. In iRBC, the release of EVs containing hAG2-hsa-miR-451a/hsa-let-7b (upper right box) fuse with the recipient EC to induce the expression of V-CAM molecules that are required to interact with iRBC through pfEMP1 on their surface. Additionally, ECs release IL-1 and IL-6, which promote vascular dysfunction. (*c*) MiRNAs during plasmodium pathogenesis in the placenta and liver. (i) Liver. Parasite infection downregulates various miRNAs associated with lipid metabolism. Alteration of the expression of some miRNAs such as hsa-miR-192 and hsa-miR-98, promotes overexpression of sterol-regulatory element-binding proteins (SRBPE1/2) and the subsequent lipid accumulation in liver tissue, which causes the downregulation of hepatocyte nuclear factor 4 alpha (HNF4-α) and hsa-miR-101 and hsa-miR-192. In addition, hsa-miR-101 downregulation induces the expression of a histone-lysine methyltransferase (EZH2) involved in the epigenetic regulation of immunological genes and the maintenance of negative feedback that keeps hsa-miR-101 downregulated. Dysregulation of miRNA during *P. chabaudi* in mice and miRNA altered during vaccination are shown at the right of (ii). (ii) Placenta. Infected cytotrophoblasts release EVs containing hsa-miR-517c (C19MC) involved in immune pathology, specifically pre-eclampsia and spontaneous abort. Genetic constitution is also relevant since homozygosity can increase parasite infection.

Like kinetoplastid parasites, *Plasmodium* species lack miRNA pathways [218,219]. However, this parasite imports the human miRNA-RISC complex and regulates its genes' expression [114,220,221] (figure 5).

In malaria, host cell miRNAs appear to play dichotomous roles where some miRNAs are associated with the pathogenesis while others confer resistance and protective immune response against this infection [222,223]. Thus, 50 miRNAs are differentially expressed (7 upregulated and 43 downregulated) in human malaria; many regulate genes involved in immune responses, including the TNF pathway and T cell receptor signalling [224].

#### 6.4.2. Cerebral malaria

MiRNAs present critical regulatory roles in cerebral malaria (CM) neuropathogenesis (figure 5*a*), promoting the persistence of infection [222,225–228]. Although the sequestration of infected erythrocytes (IEs) within cerebral blood vessels is a significant component of the pathogenesis, immune system imbalance, apoptosis and hypoxia can play important roles [229–231]. Furthermore, infections caused by different species of plasmodium induce dysregulation of a group of 12 specific miRNAs [222]. Thus, hsa-let-7i, hsa-miR-27a, hsa-miR-150, hsa-miR-19a, hsa-miR-19b, hsa-miR-142 and hsa-miR-223 are upregulated in the brain in response to *Plasmodium berghei* ANKA strain infection. Most miRNAs target genes linked to cellular proliferation, endocytosis, adherent junctions, FoxO, TGF-β and IFN-γ signalling pathways [222,225,227] (figure 5*a*). Notably, many of these signalling pathways negatively regulated by these miRNAs are part of the defence mechanism against plasmodium [232,233]. For instance, TGF-β is a fundamental regulator in the immune response against plasmodium infection, as it maintains ‘immune balance’ during infection [232]. In addition, FoxO provides integration between growth factor signalling, oxidative stress and inflammation, contributing to the redox balance in CM. Moreover, FoxO also regulates the expression of genes involved in apoptosis and glucose metabolism [233].

On the other hand, hsa-miR-146a and hsa-miR-193b are significantly more abundant in microvesicles isolated from CM mice (figure 5*a*) than in non-infected and non-CM mice. Both miRNAs play roles in inflammation, and their dysregulation during CM has been related to the development of neurological syndrome [227]. Likewise, hsa-miR-155 levels also increased in the brain and circulating EVs of CM mouse models [234]. Hsa-miR-155 regulates endothelial activation, inflammation and blood–brain barrier (BBB) dysfunction in CM; its knockdown improves survival and preservation of BBB integrity, even in high cytokine production [234]. In addition, studies provide evidence of the role that some of these miRNAs may play in the protective immune response against malaria [222].

#### 6.4.3. Non-cerebral malaria

In non-cerebral malaria, miRNAs are involved in the resistance and protective immune response against the parasite. During the intraerythrocytic life cycle of *P. falciparum*, a subset of host cell miRNAs are captured by the parasites where they negatively regulate parasite gene expression [220,221,235,236] (figure 5*b*). hsa-Let-7i/a, hsa-miR-451, hsa-miR-197, hsa-miR-15a, hsa-miR-223 and hsa-miR-19b are some of the captured miRNAs; they regulate parasite growth through inhibition of translation by impairing ribosomal loading [220,221,235,236]. The regulatory subunit of cAMP-dependent protein kinase (PKA-R), phosphoethanolamine N-methyltransferase (PEAMT) and ring-exported protein-1 are parasite genes regulated by these host miRNAs. For instance, miR-451 modulates PKA-R's expression, resulting in increased PKA catalytic activity that, in turn, facilitates parasite invasion, survival and induction of gametocytogenesis [237]. In addition, hsa-miR-197 decreases the expression of plasmodium apicortin (PfApicortin), which plays a vital role in the stabilization of microtubules [238] leading to reduced parasite growth, micronemal discharge and attenuated merozoite invasion [221] (figure 5*b*).

Moreover, AGO2 is also imported by *P. falciparum*, where it forms specific complexes with hsa-let-7a and hsa-miR-15a and targets genes of transporters (i.e. lipid/sterol: H symporter) and proteins involved in the replication of plasmodium DNA (i.e. Rad54) [220]. Like hsa-miR-451, the accumulation of hsa-miR-19b and hsa-miR-23 inside the parasite diminishes virulence and survival of the parasite [226] (figure 5*b*). It is little known what determines the specific enrichment or incorporation of specific miRNAs in the parasite. However, this mechanism could offer a potential therapeutic strategy for malaria.

In addition, erythrocyte miRNAs play an essential role in communication between the IEs, endothelial and immune cells [239,240] (figure 5*b*). IEs produce EVs containing miRNAs (EVs-miRNAs), including hsa-miR-451a and hsa-let-7b, and functional miRNA-AGO2 complexes that are internalized by ECs where they modulate the vascular function through regulation of the expression of target genes and barrier properties [240]. The EVs-miRNAs induce pro-inflammatory cytokines such as IL-6 and interleukin-1 (IL-1), which impair the endothelial barrier function and induce the expression of adhesion molecules, contributing to vascular dysfunction by excessive adhesion and transmigration of leucocytes. Additionally, the expression of transcription factor ATF2, caveolin-1 (CAV-1) and surface receptor vascular cell adhesion protein 1 (VCAM-1) is altered by the internalization of EVs by ECs [240]. Importantly, VCAM-1 is a receptor of *P. falciparum* erythrocyte membrane protein 1 (PfEMP1) that mediates IE adhesion to the endothelium [241].

The liver, an organ involved in the intracellular proliferation of the parasites during the early phase of malaria, also experiments miRNAs reprogramming [242,243] (figure 5*c*i). Thus, *Plasmodium chabaudi* (*P. chabaudi*) induces differential hepatic miRNA expression in infected mice [242,243]. In this study, 3 miRNAs (mmu-miR-26b, mcmv-miR-M23-1-5p and mmu-miR-1274a), and 16 miRNA (mmu-let-7a, mmu-miR-101b, mmu-miR-192, mmu-miR-98, mmu-miR-142-5p and mmu-miR-193a-3p, between others), were, respectively up- and downregulated [242]. Strangely, none of these 19 miRNAs are involved in the immune response [244], although the liver contains both intrahepatic and migratory B and T cells, especially during *P. chabaudi* infections [245].

Increased expression of hsa-miR-26b, mcmv-miR-M23-1-5p and hsa-miR-1274a has also been reported in response to other viral, bacterial and parasitic infections [243,246,247]. For example, an increase in the expression of hsa-miR-26b is related to an inhibition of a protective immune response against *Mycobacterium tuberculosis (M. tuberculosis)* through inhibition of the NF-κB pathway by directly targeting TGFβ-activated kinase-1, enabling the replication of *M. tuberculosis* [247]. On the other hand, the upregulation of hsa-miR-1274a has upregulated response to infection by avian influenza A H5N1 virus, where hsa-miR-1274a targets BCL2-associated transcription factor 1 and TNF alpha induced protein 3 [246]. In addition, mcmv-miR-M23-1-5p is upregulated significantly in murine infections caused by the intestinal apicomplexan *Eimeria papillate* (*E. papillat*e). Although the function of mcmv-miR-M23-1-5p is unknown, its upregulation, along with that of other miRNAs, is associated with low inflammation and strong IFN-γ response during *E. papillate* [248].

Regarding the downregulated hsa-miR-101b, hsa-miR-192, hsa-miR-98 and hsa-miR-142-5p, they have been associated, respectively, with induction of hepatic tolerance after transplantation [249], drug-induced liver injury [250,251], liver metabolism [252] and hepatocellular carcinoma [253]. The downregulation of hsa-miR-192 and hsa-miR-98 is particularly interesting since both play essential roles in altering lipid metabolism, specifically, hepatic lipid accumulation [252,254] (figure 5*c*ii). For its part, *P. chabaudi* induces an accumulation of triacylglycerol, free FA and free cholesterol in this organ through the modulation of the expression of enzymes and transcription factors involved in lipid metabolism [255], including the 5′ AMP-activated protein kinase, an essential regulator of cellular energy metabolism [255]. This manipulation of the host cell's lipid metabolism by plasmodium is essential for its development and proliferation [255]. Moreover, hsa-miR-192 and hsa-miR-98 target sterol-regulatory element-binding proteins (SREBPs), SREBP-1 and SREBP-2, respectively [252,254] (figure 5*c*i). SREBPs are a family of classical transcription factors that directly activate the expression of more than 30 genes related to the synthesis of cholesterol, FAs, triglycerides and phospholipids, in the kidney and other tissues [256]. Thus, the dysregulation of cellular lipid metabolism by Plasmodium could be a strategy to facilitate its development, proliferation and lifespan in its vertebrate host.

It should be noted that the authors [242] associate the sustained expression of this miRNA profile with the acquisition of protective immunity against *P. chabaudi* malaria and propose that epigenetic mechanisms could be involved in their differential expression. However, none of the miRNAs reported in this study has been identified as epigenetically regulated [257–260]. Instead, only hsa-miR-101 has been recognized as epi-miRNAs, a class of miRNAs that can regulate the epigenetic modifiers [261–263]. However, the transcription factor HNF4*α* (hepatocyte nuclear factor 4 alpha) essential for basal expression of liver-enriched microRNAs that include hsa-miR-101 and hsa-miR-192, is downregulated by lipids [264] (figure 5*c*i). Therefore, it is possible that plasmodium infection initially induces downregulation of miRNAs associated with the lipid metabolism leading to an accumulation of lipids which decreases the level of HNF4*α* that subsequently decreases the expression of its dependent miRNAs, including hsa-miR-101 and hsa-miR-192 [264].

On the other hand, genomic deletion and suppression of hsa-miR-101 induce overexpression of the Enhancer of Zeste homologue 2 (EZH2) [265,266], a histone-lysine methyltransferase (EZH2) that is part of the polycomb repressive complex 2 (PRC2). Studies have postulated that there is a reciprocal negative feedback loop between miR-101 and EZH2. In this EZH2-mediated regulation of the hsa-miR-101 loop, high levels of the EZH2 factor contribute to the depletion of hsa-miR-101, which, in turn, causes cells to maintain increased EZH2 levels, hence a sustained muting of hsa-miR-101 [267] (figure 5*c*i). Although EZH2 is associated with gene silencing, EZH2 has been reported to play essential roles in cell-mediated and humoral adaptive immunity during later stages of immune cell differentiation [268]. In the context of infections caused by plasmodium, the feedback loop between some miRNAs linked to lipid metabolism and EZH2 may play a key role in developing protective immunity through modifications of the epigenome.

Factors such as protective vaccination during *P. chabaudi* malaria can induce changes in the expression of some miRNA species and activate epigenetic remodelling processes regulated by them [243]. The upregulation of members of the let-7 family (mmu-let-7a-5p, mmu-let-7b-5p, mmu-let-7c-5p, mmu-let-7d-5p, mmu-let-7f-5p, mmu-let-7 g-5p and mmu-let-7i -5p), mmu-miR-26a, mmu-miR-122-5p, mmu-miR-30a, mmu-miR-27a and mmu-miR-29a is associated with liver regeneration, while the upregulation of hsa-miR-142-3p is associated with enhanced hepatic erythropoiesis, possibly at the expense of megakaryopoiesis [243]. The physiological significance of the upregulation of these miRNAs may be related to the role of accelerated liver regeneration during *P. chabaudi* infections. During the acute phase, remodelling makes it possible to deal with liver dysfunctions caused by serious lesions induced by this parasite [269].

In the placenta, *P. falciparum* infection causes changes in trophoblast extracellular vesicle miRNA (trEVs) content. Hsa-miR-517c is overexpressed in mothers with placental malaria compared to non-infected ones [270] (figure 5*c*ii). This miRNA, belonging to the C19MC cluster, has immunomodulatory functions during pregnancy [271]. Additionally, increased expression of hsa-miR-517c has been observed in placental weight [272], pre-eclampsia [36] and recurrent spontaneous abortion [273]. From the genetic point of view, polymorphisms in miRNAs can influence susceptibility to infection by plasmodium. For example, the has-miRNA-146a polymorphism increases the odds of malaria in pregnancy. Thus, homozygosity increases up to 6% the probability of placental infection [274].

#### 6.4.4. miRNA in host–*Toxoplasma gondii* interaction

In *T. gondii*, unlike the protists analysed so far, the machinery for small RNA generation and miRNA-mediated gene regulation is present [136]. In this protist, miRNAs, besides regulating the expression of their genes [275], regulate parasite–host communication [276]. These miRNAs are differentially expressed between different isolates [275,277] and act as endogenous regulatory factors that modulate cell differentiation and development [275]. One characteristic of these *T. gondii* miRNAs is that some are homologous to human/rodents' miRNAs, while others (99% miRNAs) are isolate-specific [275,276]. Also, depending on the isolate, these miRNAs can represent 5–8% of the total of the small ncRNAs [275].

**Figure 6.**
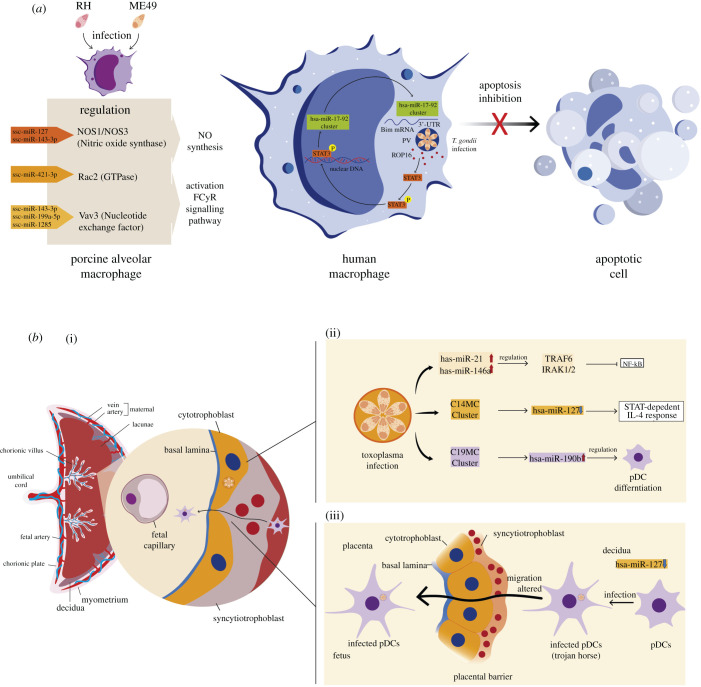
(*a*) MicroRNAs during toxoplasma pathogenesis. (*a*) Role of *T. gondii*-infected human and porcine macrophage miRNAs. During infection of porcine macrophages by toxoplasma RH/ME49, various miRNAs regulate the expression of NO synthesis and FC*γ*R signalling pathway by directly targeting mRNAs of NOS1/NOS3, Rac2 and Vav3. In human macrophages, the parasites in the PV release ROP16, a protein kinase that phosphorylates and activates the transcription factor STAT3. The activated factor travels to the nucleus and stimulates the miR-17-92 cluster; this miR cluster is responsible for post-transcriptional regulation of the mRNA of the pro-apoptotic protein ‘Bim’ through the binding to the 3′-UTR region of the mRNA molecule. This process prevents the infected macrophage from activating the apoptosis pathway during toxoplasma infection, thus allowing the survival of the parasites. (*b*)(i) Role of miRNAs in the pathogenesis of *T. gondii* in the placenta. (ii) In toxoplasma-infected placenta (cytotrophoblasts), dysregulation of immunomiRs, and the C14MC and C19MC clusters inhibit the NF-kB response and alter the STAT-dependent IL-4 response and pDC differentiation, respectively. (iii) In pDCs, the downregulation of hsa-miR-127 during toxoplasma infection elicit an altered migration by these cells, causing them to function as Trojan horses to cross the placental barrier and reach the fetus environment.

Numerous studies show that *T. gondii* infection alters the expression of host miRNAs [144,278–281] and that host survival and parasite virulence are regulated by these miRNAs [281] (figure 6*a*,*b*). Even when the infection is chronic and invasion of the central nervous system occurs, *Toxoplasma* can promote brain carcinogenesis by altering the host miRNAs [279]. This modulation can occur by altering the host's miRNA expression and exporting parasite miRNAs or hairpins to its host cell [276,280–282]. In the first case, the alteration of host miRNAs expression can occur through parasitic effector proteins (proteins of rhoptry and dense granule secretory organelles). These proteins interfere with miRNA synthesis and maturation pathways, and modulate host cell survival or death [278,283,284]. In the export of parasitic hairpins that may act as miRNAs in humans [276].

Macrophages infected with different *T. gondii* isolates present different miRNA expression profiles [280,281,283] (figure 6*a*). Thus, infection of macrophages with the Chinese 1 Ctwh3 isolate causes an increase in the expression of the miR-17–92 gene cluster, leading to the inhibition of apoptosis in the host cell. This overexpression is orchestrated by the secretory kinase ROP16 [284], a rhoptry protein and one of the major virulence factors of *T. gondii* [285]. This protein is injected into the host cell's cytoplasm during the invasion process and phosphorylates STAT transcription factors [286]. Once phosphorylated, STAT goes to the nucleus and selectively induces the transcription of a subset of miRNAs (hsa-miR-19a, hsa-miR-19b and hsa-miR-20a) that are part of the miR-17–92 gene cluster. Subsequently, the hsa-miR-17-92 induced by STAT3 binds to the 3′ UTR sequences of the Bim mRNA, promoting the reduction of the pro-apoptotic protein BIM, thus inhibiting apoptosis [278,283] (figure 6*a*). In this sense, the miR-17–92 cluster contributes to the downregulation of host cell apoptosis by inhibiting cytochrome c release and subsequent caspase activation and by decreasing (ADP-ribose) polymerase protein levels [287].

Alveolar macrophages infected with *T. gondii* strain RH (Type I) and Me49 (Type II) also show a differential expression of 89 miRNAs, many of them related to resistance and elimination of the parasite [281] (figure 6*a*). For instance, ssc-miR-127 and ssc-miR-143-3p are predicted to regulate, respectively, nitric oxide synthase 1 (NOS1) and nitric oxide synthase 3 (NOS3), enzymes that oxidize L-arginine. Furthermore, ssc-miR-421-3p regulates Rac2 small GTPase, while ssc-miR-143-3p, ssc-miR-199a-5p and ssc-miR-1285 are predicted to regulate Vav3, a guanine nucleotide exchange factor. Both are important for the activation of the Fc*γ*R signalling pathway, essential in defence against parasites through the processes of antigen recognition and phagocytosis in macrophages [281].

Modulation of host microRNA by *T. gondii* in the brain can promote the establishment of latency and the development of cancer [279,288]. The establishment of latency is promoted by the increase of mmu-miR-155-5p, mmu-miR-146a-5p, mmu-miR-142a-3p, mmu-miR-142b and mmu-miR-21a-5p as well as decrease of mmu-miR-409-5p, mmu-miR-127-3p and mmu-miR-493-5p. Importantly, targets genes of dysregulated miRNAs are involved in immune response-related signalling pathways (i.e. Rap1, MAPK and Hippo signalling pathways) and Fc gamma R-mediated phagocytosis. Other target genes involved in pathways associated with diseases, such as cancer, endocrine resistance and human T lymphotropic virus type 1 (HTLV-I) infection, were also detected [288].

Toxoplasma-dependent upregulation of the miR-17-92 cluster could be one of the mechanisms this parasite promotes tumorigenesis. The miR-17-92 cluster is associated with brain cancers since it correlates with high protein levels of members of the MYC proto-oncogene family [289] observed with infections of this parasite. Alteration of the expression of these transcription factors, dependent on the function of the Myc affected, represents an additional form by which toxoplasma induces specific alterations of host cell functions during intracellular growth [290]. Myc directly activates the transcription of the miR-17-92 cluster [291,292] and subsequently promotes the reduction of pro-apoptotic proteins inhibiting the process of apoptosis [278,283]. Additionally, Myc activates many genes associated with cell growth, including E2F genes, such as E2F1, required for the initial entry to the cell cycle from a quiescent state [293]. E2F1 expression is also increased in cells infected by *T. gondii* [294]. Paradoxically, the mir-17-92 cluster negatively modulates the E2F1 expression [291]. How *T. gondii* activates two opposing Myc-dependent pathways simultaneously is unknown. However, they may have a role in establishing the infection. Activation of both pathways is aimed at inhibiting apoptosis and progression through the S phase and into G2 / M. This could indicate the importance of the cell cycle stage of the host cell during infection [294].

Notably, the toxoplasma genome codes for endogenous miRNAs, so it is possible that parasites use these to modify the host's cellular functions [279], similarly to that observed for mammalian viruses [276,295]. Thus, it has been proposed that *T. gondii* exports endogenous miRNA, similar to human miRNA, to its host cell, modulating various physiological processes, including apoptosis [276,279]. About 150 *T. gondii* miRNAs are similar to human miRNAs, including hsa-miR-6873, hsa-miR-328, hsa-miR-7107-3p, -3p, hsa-miR-6821-5p and hsa-miR-4644 [276]. For instance, hsa-miR-4644 is overexpressed and increases cell proliferation and survival by inhibiting the expression of the antiproliferative UbiA prenyltransferase domain-containing protein 1 (UBIAD1) [296]. In addition, UBIAD1 mediates the formation of menaquinone-4 (MK-4, a vitamin K2 isoform) and coenzyme Q10 that modulate the activities of enzymes involved in mitogenesis, cell growth, neuronal protection during ischaemic/hypoxic injury, regulation of glial cells, and sphingolipid synthesis and metabolism [297]. Therefore, it is very likely that toxoplasma uses endogenous miRNAs to inhibit apoptosis and promote the establishment of infection and manipulate the defence system and some of the biosynthetic pathways necessary for its metabolic support.

*T. gondii* induces in the placenta differential expression of 42 miRNAs (16 negatively regulated and 29 positively regulated). Some are encoded in the placenta-specific C19MC and C14MC clusters [144] (figure 6*b*). Like *T. cruzi*, *T. gondii* induces a higher expression of hsa-miR-21 and hsa-miR-146a in ontological terms [144] (figure 6*b*). Hsa-miR-146a is a negative regulator of the innate immune response and can target TRAF6 and IRAK1 / 2, inhibiting the activation of transcription factor NF*κ*B [154,298]. In *T. gondii* infections, TRAF6 contributes to a host protective immunity by regulating the production of pro-inflammatory cytokine IL-12, which is essential to control the infection, and vacuole-lysosome fusion, a fundamental step during infection [299,300]. There are also reports where hsa-miR-146a is defined as a dendritic cell (DC)-relevant miRNA, a central target of this parasite [301]. For its part, hsa-miR-21 plays a role in the host response to infection by another apicomplexan. Thus, the upregulation of hsa-miR-21 in.biliary epithelial cells infected by *Cryptosporidium parvum* is associated with epithelial anti-microbial defence against this parasite [302].

Regarding placenta-specific miRNAs encoded in the C14MC cluster and downregulated by *T. gondii,* some of them, such as hsa-miR-127-3p, are associated with placental pathologies [144] (figure 6*b*). Hsa-miR-127-3p regulates the expression of genes involved in lung development, apoptosis and placental formation. In addition, hsa-miR-127-3p is involved in antisense regulation of Rtl1 imprinting. Rtl1 is a retrotransposon-derived protein-coding gene essential for maintaining fetal capillaries and the feto-maternal interface [195]. Hsa-miR-127 also targets BCL6, a transcription factor with a zinc-finger domain that regulates the expression of genes involved in the STAT-dependent IL-4 response [303]. The deregulation of BCL6 causes the imbalance between apoptosis and cell proliferation [303,304] and increases the levels of ZEB2 protein [303,305]. ZEB2 plays an important role in immune cell development and function; it acts as a fate switch between plasmacytoid dendritic cells (pDCs) and conventional dendritic cells (cDCs) [306]. Thus, overexpression of ZEB2 increases pDCs [307]. Therefore, the downregulation of hsa-miR-127 in.response to *T. gondii* may be related to the fact that pDCs play a protective role during infection since they participate in the early stages of infection through the presentation of antigens and modulation of the immune response triggered by cytokines [308,309].

Additionally, these pDCs are efficient in autophagy, eliminating the parasite in primed macrophages [310,311]. However, the ability of *T. gondii* to functionally inactivate pDCs has been reported, using them as Trojan horses to cross the placenta [301,312] (figure 6*b*). pDCs have been identified in the decidua of early human pregnancy [313]. Knowing if hsa-miR-27 downregulation occurs as a host cell response to infection by *T. gondii* or if this is a mechanism by which the parasite can induce favourable conditions to promote the spread of infection deserves further studies.

*T. gondii* also alters the expression of miRNAs encoded in the C19MC cluster (figure 6*b*). Thus, the parasite upregulates hsa-miR-190b in *ex vivo* infected HPE [144]. Hsa-miR-190b is associated with some types of cancer and viral infections [314,315]. For example, in cancer, upregulation of hsa-miR-190b promotes the inhibition of apoptosis through repression of PTEN, a protein involved in regulating the cell cycle [314]. Also, the increase of hsa-miR-190b expression in neurons suppresses autophagy and decreases pro-inflammatory TNF-α, IL-6 and IL-1*β* cytokines [316]. The target genes of this miRNA include genes involved in cell adhesion and transcription factor 4 (TCF-4) [315]. TCF-4 participates in brain and immune system development, including pDC differentiation [317]. Therefore, it is likely that the modulation of miRNAs linked to apoptotic cell death in the host represents a mechanism to avoid rapid clearance. The modulation of some specific placental miRNAs suggests a mechanism associated with the manipulation of the immune response, which could even involve the promotion of a line of DC, which are functionally sequestered by this parasite.

The *T. gondii* effect on host miRNAs is not strictly tissue-specific. Thus, hsa-miR-155-5p and hsa-miR-21-5p modulation has been reported in the spleen and eyes [318,319]. Even in AIDS / cerebral toxoplasmosis co-infected patients, hsa-miR-21-5p is upregulated [320]. Hsa-miR-21 is a crucial mediator for the inflammatory response since it regulates the anti-inflammatory cytokine IL-10 and TNF-α production levels. The regulation of these cytokines is essential for the balance and transition between symptomatic and asymptomatic infection [320].

In summary, the infection caused by the apicomplexans plasmodium and toxoplasma is characterized by inducing changes in the expression of host miRNAs. Unlike what is observed in kinetoplastids, the expression pattern of most of these non-coding RNAs is more heterogeneous since it depends on the species and isolates of the parasite and the tissues involved. Notably, some of these host-modulated miRNAs are oncogenic, leading to the development of some types of cancer. Moreover, deregulated miRNAs in response to infection by these pathogens (e.g. -miR-146 family, hsa-miR-155 and has-miR-21) also undergo modifications in their expression in infections caused by kinetoplastids.

## 7. Conclusion and possible trends for future research

**Figure 7.**
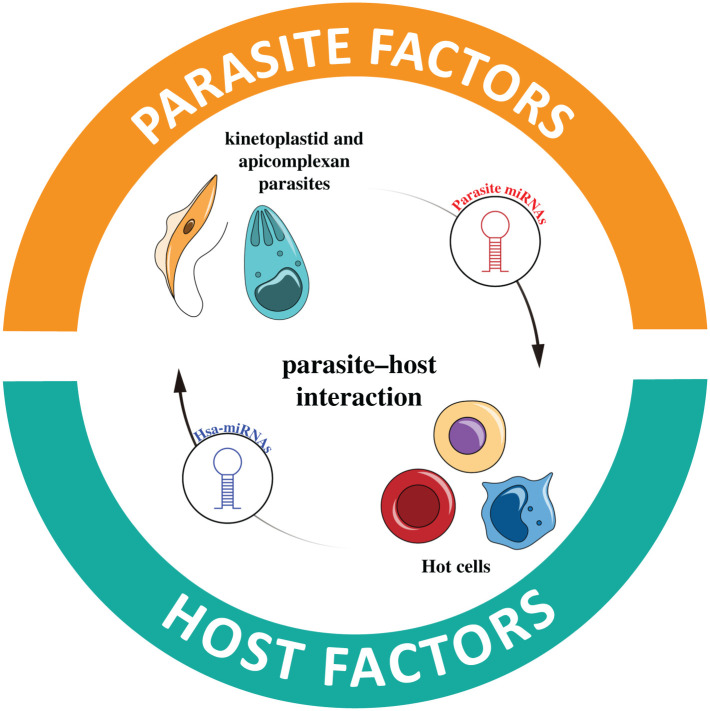
MiRNAs and their role in parasite–host interaction: MiRNAs modulate parasite and host responses during parasite infection, determining disease probability. In the parasite, miRNAs modulate cellular proliferation, differentiation, metabolism and drug resistance. In the host, miRNAs play essential roles in host defence mechanisms.

MiRNAs are small non-coding RNAs distributed in a wide variety of organisms and are relevant during host–pathogen interactions, modulating many biological aspects in the pathogen and the host cells (figure 7).

Moreover, miRNAs are considered as promising diagnostic and prognostic tools as well as treatment targets for different pathologies, including infections [321]. Thus, changes in miRNAs expression in pathologies are detectable in biological fluids and some of them are tissue specific [322]. Moreover, miRNAs can be modulated in multiple ways, either at the level of biogenesis or by adjusting their mode of action. Strategies for intervening biogenesis include the use of small-molecule drugs, miRNAs sponges, oligonucleotide therapies (miRNA replacement and antisense oligonucleotides) between others. In addition, different miRNA delivery systems are currently studied and developed such as biomimetic delivery systems and synthetic nanoparticles [323]. Additionally, miRNAs can be administrated *in vivo* and present an apparent lack of adverse events when administered intravenously [322]. Moreover, there are several clinical trials ongoing [324]. Thus, studies regarding the role of miRNAs during host–parasite interaction should lead to new prognostic, diagnostic and treatment possibilities.
